# Systemic immune-inflammation index and the short-term mortality of patients with sepsis: A meta-analysis

**DOI:** 10.17305/bb.2024.11494

**Published:** 2024-12-27

**Authors:** Lingbo Liang, Qiaoli Su

**Affiliations:** 1 General Practice Ward/International Medical Center Ward, General Practice Medical Center, West China Hospital, Sichuan University, Chengdu, China

**Keywords:** Sepsis, systemic immune-inflammation index, SII, prognosis, mortality, meta-analysis

## Abstract

The systemic immune-inflammation index (SII) is a novel biomarker that reflects the balance between the host immune response and inflammation, two key pathophysiological processes involved in sepsis. This meta-analysis aimed to evaluate the relationship between SII at admission and short-term mortality in patients with sepsis. Literature searches were performed in PubMed, Embase, Web of Science, CNKI, and Wanfang up to August 30, 2024, using relevant search terms. Observational studies that reported the association between SII and short-term mortality in sepsis patients were included. Risk ratios (RRs) and 95% confidence intervals (CIs) comparing the incidence of mortality within 90 days in patients with sepsis with a high versus low SII were calculated. Nine cohort studies, with a total of 25,626 patients, were included. A high SII at admission was significantly associated with an increased risk of all-cause short-term mortality in sepsis patients (RR: 1.51, 95% CI: 1.31–1.67, *P* < 0.001), with moderate heterogeneity (*I^2^* ═ 43%). Sensitivity analyses confirmed the robustness of these findings. Subgroup analyses suggested a stronger association in patients younger than 67 years compared to those aged 67 years or older (*P* ═ 0.04), but no significant differences were observed based on sex, SII cutoff values, or follow-up duration. In conclusion, this meta-analysis demonstrates that elevated SII at admission is associated with an increased risk of short-term mortality in sepsis patients, particularly in younger individuals. Further research is needed to validate these findings and explore their clinical implications.

## Introduction

Sepsis, a life-threatening organ dysfunction resulting from a dysregulated host response to infection, remains one of the leading causes of morbidity and mortality globally [1–3]. Despite significant advancements in early diagnosis and treatment, the incidence of sepsis has risen substantially, with an estimated 49 million cases and over 11 million related deaths annually worldwide [4]. The high mortality rate, particularly among critically ill patients, underscores the importance of early identification and risk stratification [5]. Currently, risk stratification models, such as the Sequential Organ Failure Assessment (SOFA) score [6] and the Acute Physiology and Chronic Health Evaluation (APACHE) score [7] are widely used to predict mortality in septic patients. However, these models often require detailed clinical and laboratory data, making them time-consuming and complex to implement in real-time clinical settings [8, 9]. Additionally, their ability to accurately predict short-term mortality is limited by variability in patient populations, clinical settings, and the timing of assessments [8, 9]. Thus, there is a critical need to identify novel, convenient, and reliable biomarkers to improve early risk stratification and guide clinical decision-making for patients with sepsis.

One such emerging biomarker is the systemic immune-inflammation index (SII), a novel prognostic factor that reflects the balance between immune response and inflammation [10]. The SII is calculated using a simple formula based on routine complete blood count (CBC) parameters: platelet count × neutrophil count/lymphocyte count [11, 12]. This simplicity allows SII to be calculated from standard laboratory tests, facilitating its widespread use in various clinical settings without the need for specialized equipment or additional testing [10]. Pathologically, an elevated SII indicates heightened immune activation, increased inflammatory response, and dysregulation of the coagulation system, all of which play critical roles in the pathophysiology of sepsis [13]. These processes contribute to endothelial dysfunction, microvascular thrombosis, and multi-organ failure, leading to poor clinical outcomes [14]. The convenience of calculating SII and its potential to reflect both immune and inflammatory responses make it an attractive candidate for prognostic assessment in septic patients [15]. Current research has started to explore the relationship between SII and mortality in sepsis, with some studies suggesting that elevated SII is associated with higher short-term mortality. However, these studies vary in sample sizes, populations, and methodologies, and the overall strength and consistency of this association remain unclear [16–24]. Despite growing evidence from individual studies suggesting a prognostic role of SII in sepsis, no meta-analysis has yet synthesized this evidence to evaluate its association with short-term mortality or other sepsis-related outcomes. Given the potential of SII as a convenient and powerful prognostic marker, this meta-analysis aims to systematically evaluate the association between elevated SII at admission and the short-term mortality risk in patients with sepsis.

## Materials and methods

The study adhered to PRISMA 2020 [25, 26] and the Cochrane Handbook for Systematic Reviews and Meta-analyses [27] guidelines for conducting this meta-analysis, including study design, data collection, statistical analysis, and results interpretation. The study protocol has been registered at PROSPERO (https://www.crd.york.ac.uk/prospero) with the identifier CRD42024598895.

### Literature search

To identify studies pertinent to this meta-analysis, we searched the PubMed, Embase, Web of Science, Wanfang, and China National Knowledge Infrastructure (CNKI) databases using an extensive array of search terms, which included: (1) “systemic immune-inflammation index” OR “SII” OR “systemic immune inflammation index”; and (2) “sepsis” OR “septicemia” OR “septic”. The search was limited to research involving human subjects, and we included only studies published in English or Chinese. The detailed search strategy for each database is shown in Supplementary data. Additionally, we manually reviewed the references of relevant original and review articles to identify further pertinent studies. The literature was assessed from the inception of the searched databases up to August 30, 2024.

### Inclusion and exclusion criteria

The inclusion criteria for potential studies were defined according to the PICOS framework:

- P (Population): Adult patients (aged 18 years or older) with a confirmed diagnosis of sepsis.
- I (Exposure): SII was measured and calculated within 72 h after admission, with a high level of SII at admission considered as exposure. The methods for determining the SII cutoff were consistent with those used in the original studies.
- C (Comparison): Patients without a low level of SII at admission were considered as controls.
- (Outcome): Incidence of all-cause mortality within 90 days, compared between patients with high vs low levels of SII at admission.
- S (Study Design): Observational studies with longitudinal follow-up, such as cohort studies, nested case-control studies, and post-hoc analyses of clinical trials.

The exclusion criteria included reviews, editorials, meta-analyses, preclinical studies, cross-sectional studies, studies involving patients with diagnoses other than sepsis, studies not evaluating SII as an exposure, or studies that did not report the incidence of short-term all-cause mortality. If two or more studies with overlapping populations were identified, the study with the largest sample size was included in the meta-analysis.

### Study quality evaluation and data extraction

The literature search, study identification, quality assessment, and data extraction were conducted independently by two authors. Any disagreements regarding study inclusion were resolved through detailed discussion and mutual consensus. If consensus could not be reached, the issue would have been documented and addressed in consultation with a third-party expert; however, such a situation did not arise in this study. Study quality was evaluated using the Newcastle–Ottawa scale (NOS) [28], which assesses selection, control of confounders, and outcome measurement and analysis, with scores ranging from 1 to 9, where nine signifies the highest quality. The data collected for analysis included study details (author, year, country, and design), participant characteristics (diagnosis, sample size, age), timing of SII measurement, methods for determining the cutoff values of SII, cutoff values defining a high SII, follow-up durations, number of patients who died during follow-up, and the variables adjusted for when analyzing the association between SII and short-term mortality in patients with sepsis.

### Statistical analysis

The association between SII at admission and the risk of short-term mortality in patients with sepsis was analyzed using risk ratios (RRs) and 95% confidence intervals (CIs), comparing patients with high vs low SII at admission. For studies that provided odds ratios (ORs), we converted these to RRs using the formula: RR ═ OR / ([1-pRef] + [pRef × OR]), where pRef is the prevalence of the outcome in the reference group (patients without a low TT) [29]. The RR values and their standard errors were computed from 95% CIs or *P* values and logarithmically transformed for variance stabilization. To assess heterogeneity, we used the Cochrane Q test and *I^2^* statistics [30], with *I^2^* > 50% indicating significant statistical heterogeneity. A random-effects model was applied to integrate the results, accounting for study variability [27]. Sensitivity analyses were performed by excluding individual studies sequentially to evaluate the robustness of the findings. Additionally, a sensitivity analysis limited to studies with multivariate analyses was also performed. Predefined subgroup analyses were performed to explore the effects of various factors, such as the diagnosis of the patients, mean age, sex, SII cutoff values, follow-up duration, and NOS scores. Subgroups were defined using the median values of continuous variables. Publication bias was evaluated using funnel plots and visual inspection for asymmetry, supplemented by Egger’s regression test [31]. Analyses were performed using RevMan (Version 5.1; Cochrane Collaboration, Oxford, UK) and Stata software (Version 17.0; Stata Corporation, College Station, TX, USA).

## Results

### Study inclusion

**Figure 1.**
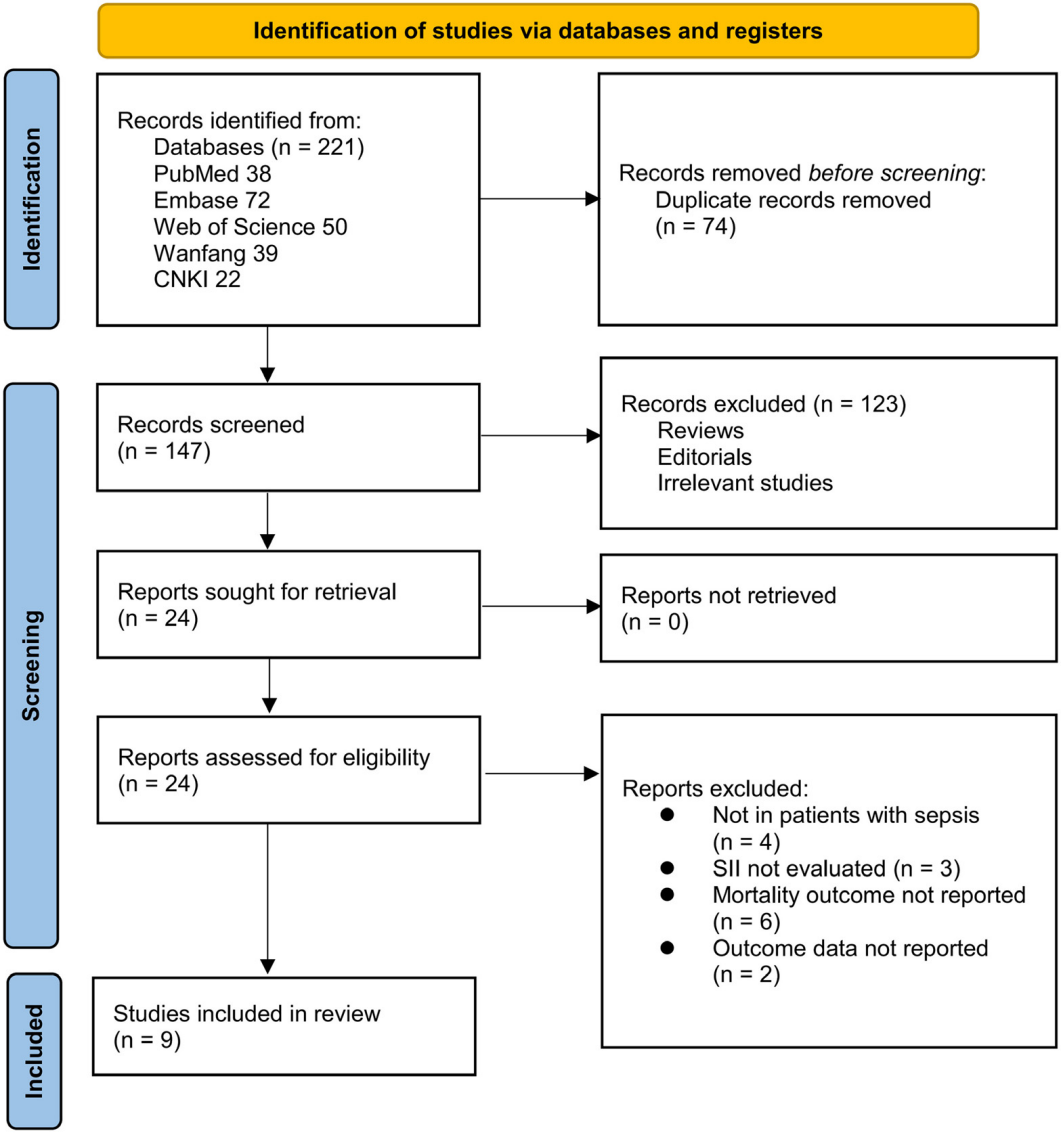
PRISMA flowchart of database search and study inclusion.

The study inclusion process is illustrated in Figure 1. Initially, 221 potentially relevant records were identified from the three searched databases, with 74 excluded due to duplication. Subsequent screening of the titles and abstracts led to the exclusion of 123 studies, primarily because they did not align with the objectives of the meta-analysis. The full texts of the remaining 24 records were reviewed by two independent authors, resulting in the exclusion of 15 more studies for various reasons, as detailed in Figure 1. Finally, nine cohort studies were included and deemed appropriate for inclusion in the quantitative analysis (16–24).

### Overview of the study characteristics

Table 1 shows the summarized characteristics of the available studies included in the meta-analysis. Overall, one prospective [21] and eight retrospective [16–20, 22–24] cohort studies were included. These studies were published from 2022 to 2024, and were conducted in China [16, 18, 20–22, 24], the United States [17, 23], and India [19], respectively. Patients with overall sepsis were included in six studies [17–21, 24], while the other three studies included patients with septic shock [22] and patients with sepsis-related acute kidney injury (AKI) [16, 23]. The diagnosis of sepsis was based on the Sepsis-3 definition in all studies except one [24], which used the Sepsis-2 definition. Overall, 25,626 patients with sepsis were included. The mean ages of the patients ranged from 49.4 to 76.8 years, and the proportions of men ranged from 56.1% to 63.0%. The SII was measured for all patients within 48 h of admission. The cutoff for defining a high SII was based on the fourth quartile of SII in two studies [17, 23] and derived using receiver operating characteristic (ROC) curve analysis in the other seven studies [16, 18–22, 24]. The cutoff values for SII varied from 535 to 3453. The follow-up duration was within hospitalization in two studies [19, 21], 28 days in six studies [17, 18, 20, 22–24], and 90 days in one study [16]. Overall, 4579 (17.9%) patients died within 90 days. Univariate analysis was performed in one study when the association between SII and mortality was evaluated [24], while multivariate analysis was performed in the other eight studies [16–23] with the adjustment of age, sex, comorbidities, and the Sequential Organ Failure Assessment (SOFA) score or the APACHE II score etc. to a varying degree. The NOS scores of the included studies ranged from six to nine, suggesting an overall moderate to good study quality (Table 2).

**Table 1:** Characteristics of the included studies

**Study**	**Country**	**Study design**	**Diagnosis**	**Definition of sepsis**	**Sample size**	**Mean age (years)**	**Male (%)**	**Timing of SII measurement**	**Methods for determining the cutoff of SII**	**Cutoff for defining a high SII**	**Follow-up duration**	**Patients died**	**Variables matched or adjusted**
Yin, 2022	China	RC	Sepsis and AKI on CRRT	Sepsis-3	90	61.3	60	24 hours within admission	ROC curve analysis derived	1730	90 days	30	Age, CRP, ALB, SOFA score, and APACHE II score
Jiang, 2023	USA	RC	Patients with sepsis	Sepsis-3	16007	67.3	57.3	24 hours within admission	Q4	3453	28 days	2110	Age, sex, comorbidities, RR, T, MAP, HR, SpO2, HGB, TB, ALB, BUN, serum glucose, anion gap, lactate, SCr, vasopressors, invasive ventilation, and CRRT
Liu, 2023	China	RC	Patients with sepsis	Sepsis-3	349	76	62.5	At admission	ROC curve analysis derived	1767	28 days	95	Age, sex, BMI, PLT, SOFA score, and APACHE II score
Mangalesh, 2023	India	RC	Patients with sepsis	Sepsis-3	267	68.1	61.4	At admission	ROC curve analysis derived	564	Inhospital	76	Age, sex, SOFA Score, CCI, length of ICU stay, and LA
Zhang, 2023	China	RC	Patients with sepsis	Sepsis-3	396	76.8	60.9	At admission	ROC curve analysis derived	935	28 days	104	Age, sex, PCT, SOFA score, and APACHE II score
Cui, 2024	China	PC	Patients with sepsis	Sepsis-3	278	57.8	56.1	At admission	ROC curve analysis derived	872	Inhospital	99	Age, sex, comorbidities, MV, LA, and SOFA score
Zhou, 2024	China	RC	Patients with sepsis	Sepsis-2	183	49.4	58.5	At admission	ROC curve analysis derived	1169	28 days	55	None
Li, 2024	China	RC	Patients with septic shock	Sepsis-3	200	63.2	63	At admission	ROC curve analysis derived	535	28 days	67	Age, sex, PCT, CRP, SCr, SOFA score, and APACHE II score
Sun, 2024	USA	RC	Sepsis and AKI	Sepsis-3	7856	66.9	57.7	48 hours within admission	Q4	3248	28 days	1943	Age, sex, comorbidities, BUN, SCr, serum glucose, serum LA, bicarbonate, potassium, vasopressor, MV, CRRT, AKI stage, SOFA, and SAP SII scores

AKI: Acute kidney injury; ALB: Albumin; APACHE II: Acute Physiology and Chronic Health Evaluation II; BMI: Body mass index; BUN: Blood urea nitrogen; CCI: Charlson Comorbidity Index; CRP: C-reactive protein; CRRT: Continuous renal replacement therapy; HGB: Hemoglobin; HR: Heart rate; ICU: Intensive care unit; LA: Lactic acid; MAP: Mean artery pressure; MV: Mechanical ventilation; PCT: Procalcitonin; PC: Prospective cohort; PLT: Platelet count; Q: Quartile; RC: Retrospective cohort; ROC: Receiver operating characteristic; RR: Respiratory rate; SAPS II: Simplified Acute Physiology Score II; SCr: Serum creatinine; SII: Systemic immune-inflammatory index; SOFA: Sequential Organ Failure Assessment; SpO_2_: Peripheral capillary oxygen saturation; T: Temperature; TB: Total bilirubin.

**Table 2:** Study quality evaluation via the Newcastle-Ottawa scale

**Study**	**Representativeness of the exposed cohort**	**Selection of the non-exposed cohort**	**Ascertainment of exposure**	**Outcome not present at baseline**	**Control for age**	**Control for other confounding factors**	**Assessment of outcome**	**Enough long follow-up duration**	**Adequacy of follow-up of cohorts**	**Total**
Yin, 2022	0	1	1	1	1	1	1	1	1	8
Jiang, 2023	0	1	1	1	1	1	1	1	1	8
Liu, 2023	1	1	1	1	1	1	1	1	1	9
Mangalesh, 2023	0	1	1	1	1	1	1	0	1	7
Zhang, 2023	0	1	1	1	1	1	1	1	1	8
Cui, 2024	1	1	1	1	1	1	1	0	1	8
Zhou, 2024	0	1	1	1	0	0	1	1	1	6
Li, 2024	0	1	1	1	1	1	1	1	1	8
Sun, 2024	0	1	1	1	1	1	1	1	1	8

### Results of the meta-analysis and sensitivity analysis

**Figure 2.**
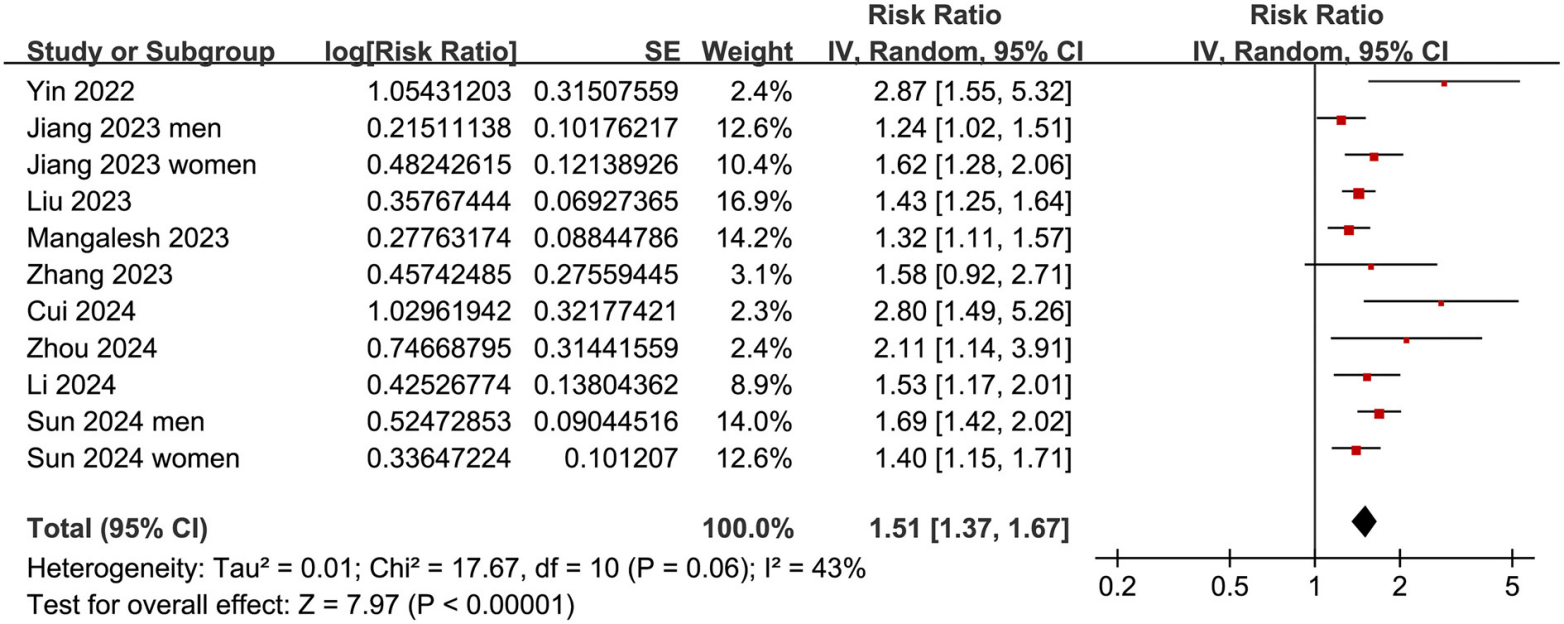
**Forest plots for the meta-analysis of the association between SII at admission and the risk of short-term mortality in patients with sepsis.** SII: Systemic immune-inflammation index.

Since two studies reported data separately for men and women [17, 23], these data were independently included, resulting in 11 datasets for the meta-analysis. The pooled results showed that a high SII at admission was significantly related with an increased risk of all-cause mortality in patients with sepsis (RR: 1.51, 95% CI: 1.31–1.67, *P* < 0.001; *I^2^* ═ 43%; Figure 2). Sensitivity analyses, performed by excluding one dataset at a time, did not significantly change the results (RR: 1.48–1.55, *P* < 0.05 for all). Notably, further sensitivity analysis limited to the eight studies [16–23] with multivariate analyses also showed similar results (RR: 1.50, 95% CI: 1.35–2.66, *P* < 0.001; *I^2^* ═ 45%).

### Results of the subgroup analyses

**Figure 3.**
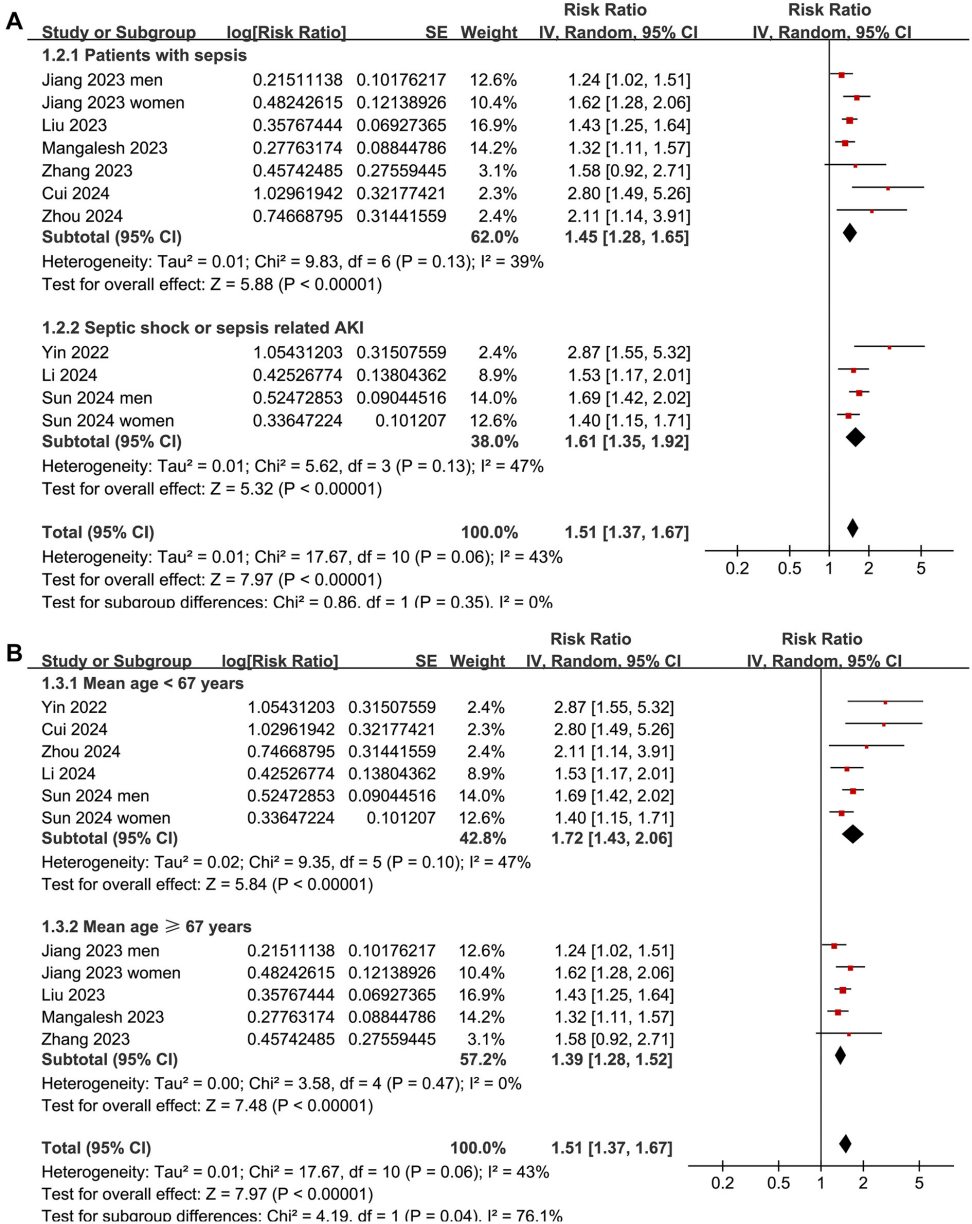
**Forest plots for the subgroup analyses of the association between SII at admission and the risk of short-term mortality in patients with sepsis.** (A) Subgroup analysis according to the diagnosis of the patients; (B) Subgroup analysis according to the mean age of the patients. SII: Systemic immune-inflammation index.

**Figure 4.**
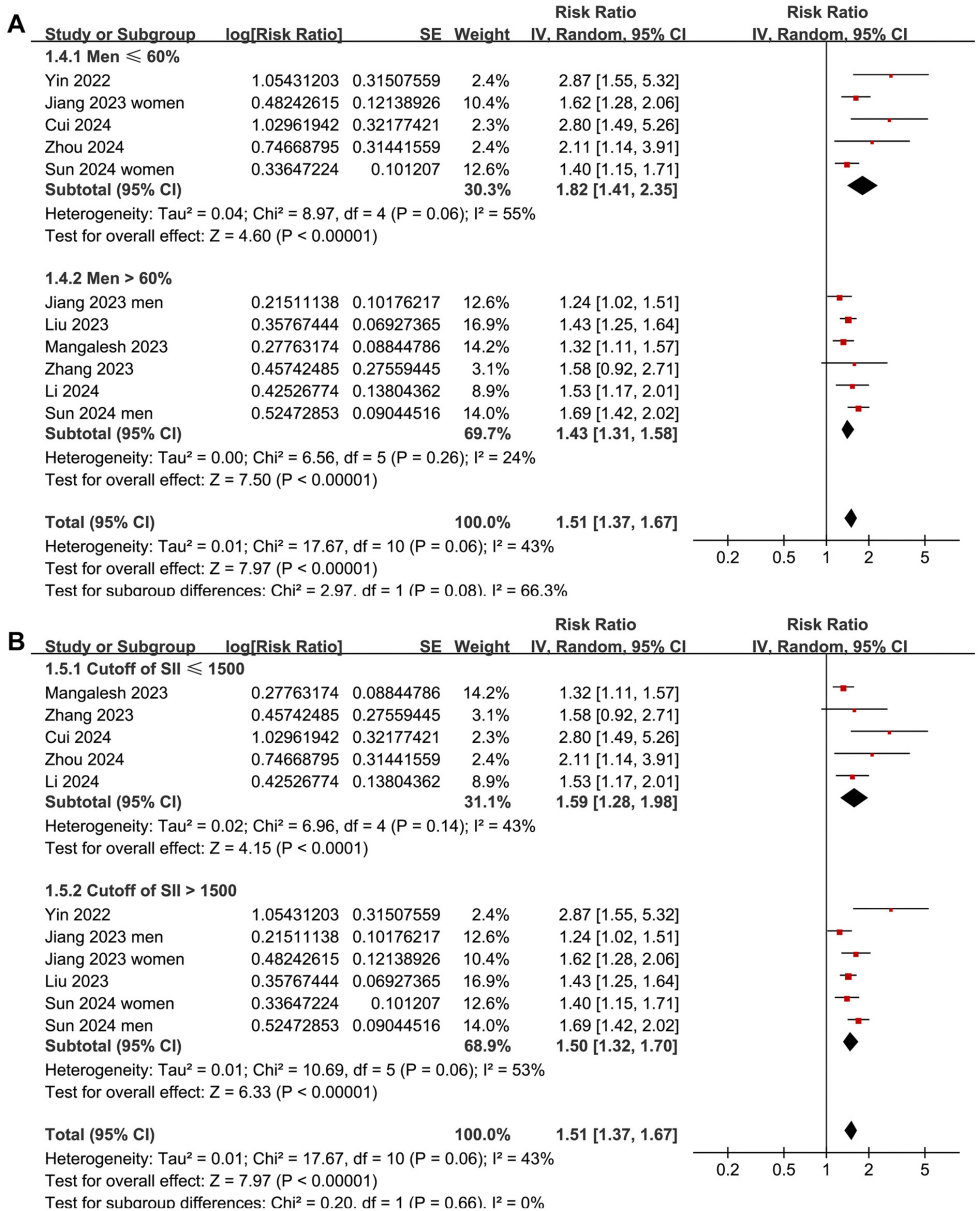
**Forest plots for the subgroup analyses of the association between SII at admission and the risk of short-term mortality in patients with sepsis.** (A) Subgroup analysis according to the proportion of men; (B) Subgroup analysis according to the cutoff of SII. SII: Systemic immune-inflammation index.

**Figure 5.**
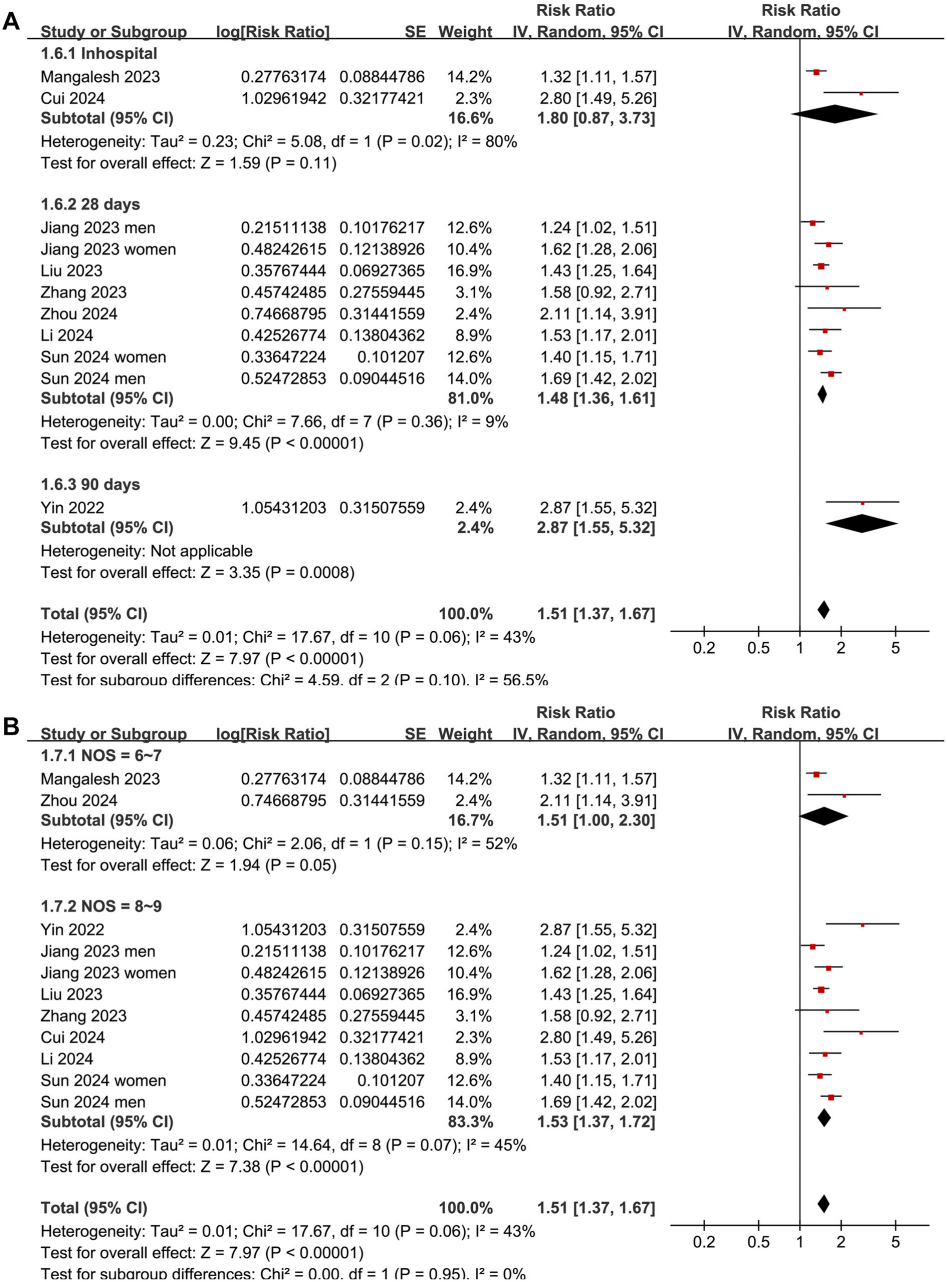
**Forest plots for the subgroup analyses of the association between SII at admission and the risk of short-term mortality in patients with sepsis.** (A) Subgroup analysis according to follow-up duration; (B) Subgroup analysis according to the NOS scores. SII: Systemic immune-inflammation index; NOS: Newcastle-Ottawa scale.

Subgroup analyses indicated that the association between a high SII and increased risk of short-term mortality was consistent among overall patients with sepsis and those with septic shock or sepsis-associated AKI (*P* for subgroup difference ═ 0.35; Figure 3A). Interestingly, it was suggested that the association between a high SII and increased risk of short-term mortality in patients with sepsis was stronger in those with a mean age < 67 years compared to those ≥ 67 years (RR: 1.72 vs 1.39, *P* for subgroup difference ═ 0.04; Figure 3B). Further subgroup analyses showed similar associations between SII and mortality risk across studies with the proportion of men ≤ or > 60% (*P* for subgroup difference ═ 0.08; Figure 4A), studies with an SII cutoff ≤ or > 1500 (*P* for subgroup difference ═ 0.66; Figure 4B), studies with different follow-up durations (*P* for subgroup difference ═ 0.10; Figure 5A), and studies with different NOS scores (*P* for subgroup difference ═ 0.95; Figure 5B).

### Publication bias

**Figure 6.**
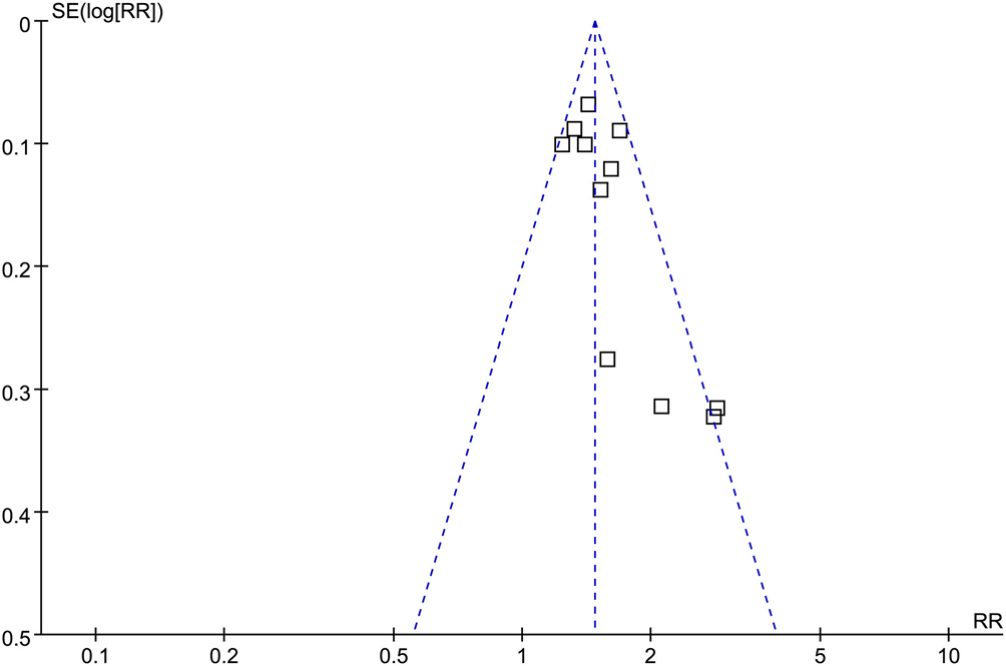
**Funnel plots for the meta-analysis of the association between SII at admission and the risk of short-term mortality in patients with sepsis.** SII: Systemic immune-inflammation index.

Upon visual inspection, the funnel plots for the meta-analysis of the association between SII at admission and the risk of all-cause mortality in patients with sepsis showed symmetry, indicating a low likelihood of publication bias (Figure 6). Additionally, Egger’s regression test results (*P* ═ 0.25) supported this conclusion, suggesting a low risk of publication bias.

## Discussion

This meta-analysis provides pilot evidence that an elevated SII at admission is significantly associated with an increased risk of short-term mortality in patients with sepsis. Our analysis, which pooled data from nine cohort studies and included over 25,000 patients, demonstrated that individuals with a high SII had a 51% higher risk of death within 90 days compared to those with lower SII levels. Importantly, this association persisted across various sensitivity and subgroup analyses, underscoring the robustness and consistency of our findings. The sensitivity analysis, limited to studies that conducted multivariate analyses, also confirmed the relationship, even after adjusting for potential confounders. Notably, the subgroup analyses revealed a stronger association between elevated SII and mortality in younger patients, suggesting that younger populations with sepsis may experience a more pronounced inflammatory response and worse outcomes when SII is elevated.

The SII is a composite biomarker that integrates neutrophil, lymphocyte, and platelet counts, each playing a critical role in the immune response to sepsis [32]. Neutrophils, as key effectors of the innate immune response, are rapidly mobilized during infection and contribute to pathogen clearance through the release of reactive oxygen species and pro-inflammatory cytokines [33]. However, in sepsis, excessive neutrophil activation can lead to tissue damage and organ failure [34]. Elevated neutrophil counts, reflected by a high SII, may indicate an exaggerated inflammatory response that overwhelms the host’s defenses, leading to poor outcomes [35]. Lymphocytes, on the other hand, are essential for adaptive immunity, and lymphopenia, represented by low lymphocyte counts in a high SII, suggests immune suppression and an impaired ability to mount an effective immune response [36]. This immune exhaustion can contribute to secondary infections and delayed recovery in sepsis patients, thereby increasing mortality risk [37]. Platelets, beyond their traditional role in coagulation, interact with neutrophils to form neutrophil extracellular traps (NETs), which have been implicated in microvascular thrombosis and organ dysfunction in sepsis [38]. Thus, a high SII reflects a combination of heightened inflammation, immune suppression, and a prothrombotic state, all of which likely contribute to the increased short-term mortality observed in sepsis patients with elevated SII levels [15].

The results of the subgroup analyses offer additional insights into the SII-mortality relationship. Interestingly, the association between high SII and mortality risk appeared stronger in younger patients, suggesting that age may modulate the inflammatory response in sepsis. Younger patients may experience a more vigorous immune response, which, when dysregulated, could lead to more severe tissue damage and worse outcomes [39]. In contrast, older patients may have a less pronounced inflammatory response, potentially due to immunosenescence or comorbid conditions, which could attenuate the effect of elevated SII on mortality [40]. Furthermore, the subgroup analysis showed that the association between high SII and mortality was consistent across different sepsis subtypes, including septic shock and sepsis-associated AKI, indicating that the SII is a reliable predictor of mortality across various sepsis presentations. The lack of significant differences in mortality risk based on the SII cutoff value, sex distribution, or follow-up duration further supports the generalizability of the findings across different clinical settings.

One of the strengths of this meta-analysis is the inclusion of a large and diverse patient population, which enhances the generalizability of the results. Additionally, the use of multivariate analyses in most of the included studies provides reassurance that the observed association between SII and mortality is independent of other confounding factors, such as age, comorbidities, and severity of illness. The comprehensive search strategy, which included multiple databases and manual reference checks, ensures that the analysis captures the most up-to-date evidence on this topic. The consistency of the results across sensitivity and subgroup analyses also strengthens the validity of our findings. However, several limitations should be acknowledged. First, most of the included studies were retrospective in nature, which may introduce the potential for selection bias and residual confounding [41]. Retrospective studies may be more prone to incomplete data collection and unmeasured confounders, which could affect the accuracy of the reported associations [42]. Moreover, the majority of the included studies were conducted in China, which may limit the generalizability of the findings to other populations, particularly in non-Asian countries where differences in healthcare systems, patient characteristics, and sepsis management may influence outcomes. Finally, the variation in SII cutoff values across studies poses a challenge in defining a universal threshold for clinical practice. While some studies used the fourth quartile of SII to define high levels, others relied on ROC curve analyses, leading to cutoff values ranging from 535 to 3453. Standardizing the definition of high SII could enhance its utility as a prognostic marker in clinical settings.

From a clinical perspective, the findings of this meta-analysis highlight the potential utility of SII as a simple and readily available biomarker for risk stratification in patients with sepsis. Given that the components of SII (neutrophils, lymphocytes, and platelets) are routinely measured in CBCs, calculating SII could provide clinicians with valuable prognostic information without requiring additional testing [43]. Identifying patients at high risk of mortality based on SII levels could help guide treatment decisions, such as the initiation of more aggressive therapies or closer monitoring in intensive care units. Furthermore, SII could be incorporated into existing sepsis severity scores, such as SOFA or APACHE II, to improve their predictive accuracy [21]. Future research should focus on validating the use of SII in prospective, multicenter studies across diverse populations. Additionally, studies investigating the effects of interventions that target the components of SII, such as immunomodulatory therapies or antiplatelet agents, could provide insights into whether modulating SII levels could improve outcomes in sepsis patients [44].

## Conclusion

In conclusion, this meta-analysis demonstrates that an elevated SII at admission is associated with significantly increased short-term mortality in patients with sepsis. The combination of neutrophilia, lymphopenia, and thrombocytosis, as captured by the SII, reflects a dysregulated immune response that drives poor outcomes in these patients. Despite the limitations of the included studies, the consistency of our findings across sensitivity and subgroup analyses suggests that SII could serve as a valuable prognostic marker in clinical practice. Future research should aim to confirm these findings in prospective studies and explore potential therapeutic interventions targeting the components of SII to improve the survival of sepsis patients.

## Data Availability

All the data generated during the study was within the manuscript and the supplemental material.

## Supplemental data

Detailed search strategy for each database:

- **PubMed (*n* ═ 38)**

((“systemic immune-inflammation index”[tw] OR “SII”[tw] OR “systemic immune inflammation index”[tw]) AND (“sepsis”[MeSH Terms] OR “sepsis”[tw] OR “septicemia”[tw] OR “septic”[tw]))

- **Embase (*n* ═ 72)**

((“systemic immune-inflammation index”:ab,ti OR “SII”:ab,ti OR “systemic immune inflammation index”:ab,ti) AND (“sepsis”/exp OR “septicemia”:ab,ti OR “septic”:ab,ti))

- **Web of Science (*n* ═ 50)**

TS=(“systemic immune-inflammation index” OR “SII” OR “systemic immune inflammation index”) AND TS ═ (“sepsis” OR “septicemia” OR “septic”)

- **Wanfang (n ═ 39)**

(“” OR “SII” OR “”) AND (“” OR “” OR “”)

- **CNKI (*n* ═ 22)**

(“” OR “SII” OR “”) AND (“” OR “” OR “”)
